# Impact of Oral Nutrition Supplements in Gastrointestinal Cancer Patients: A Randomized Controlled Trial

**DOI:** 10.3390/pharmaceutics17111443

**Published:** 2025-11-08

**Authors:** Rania M. Sarhan, Marian S. Boshra, Al Shaimaa Ibrahim Rabie, Nada A. Alzunaidy, Alzhraa M. Fahmy, Ahmed Hassan Shabaan, Hoda Rabea

**Affiliations:** 1 Clinical Pharmacy Department, Faculty of Pharmacy, Beni-Suef University, Beni-Suef 62521, Egypt; raniamohammad87@yahoo.com (R.M.S.); mariansobhy31@yahoo.com (M.S.B.);; 2 Clinical Pharmacy Department, Fayium Oncology Center, Fayium 63511, Egypt; 3 Clinical Nutrition Department, General Health Insurance Authority Fayium, Fayium 63511, Egypt; 4 Department of Food Science and Human Nutrition, College of Agriculture and Food, Qassim University, Buraydah 51452, Saudi Arabia; 5 Tropical Medicine and Infectious Diseases Department, Faculty of Medicine, Beni-Suef University, Beni-Suef 62514, Egypt; 6 Clinical Oncology Department, Faculty of Medicine, Beni-Suef University, Beni-Suef 62514, Egypt

**Keywords:** nutritional status, oral nutritional supplement, gastrointestinal cancer, patient-generated subjective global assessment (PG-SGA), whole-body composition

## Abstract

**Background**: Malnutrition is a significant national health problem in countries with low and intermediate incomes and was announced in the United Nations’ 2030 Agenda for Sustainable Development Goals. Chemotherapy may have adverse effects on nutritional health issues and quality of life experience, particularly in patients with gastrointestinal (GIT) cancer. Our research’s objective was to determine the beneficial effects of oral nutrition supplements on nutritional status assessed by maintenance of whole-body composition and patient-generated subjective global assessment (PG-SGA) in all GIT cancer patients treated with chemotherapy medications. **Methods**: Among the patients, the nutrition support (NS) group (*n* = 75) received 500 kcal daily of a balanced oral nutrition supplement formula for 12 weeks, while the control (C) group (n = 75) did not. Anthropometric measures, whole-body composition, nutritional status biomarkers, and the PG-SGA questionnaire were assessed. Additionally, this study analyzed whole-body composition, skeletal mass, fat mass, laboratory data, the complete lipid profile, albumin, total protein, adverse effects, and therapy delays. **Results:** After 12 weeks, the NS group showed a significant increase in body weight, with a mean difference of 1.27 ± 3.39, while the C group showed a mean difference of only 0.15 ± 0.42. Moreover, fat mass increased in the NS group, showing a mean difference of 0.55 ± 3.69, while the C group showed a fat mass loss with a mean difference of −0.21 ± 2.93. The fat mass index (FMI) indicated statistical significance between the two groups. There was a statistically significant difference in the lean mass index between the two groups, favoring a steady increase in the NS group. The NS group showed improvements in the PG-SGA and nutritional biochemical markers, such as albumin. The initial findings from our study include data from a total of 150 patients, including 75 patients in the NS group and 75 patients in the C group. These results are consistent with earlier research. **Conclusions:** Early oral nutrition supplements for GIT cancer may enhance nutritional outcomes and reduce the delay of disease-related therapy. Additionally, they may help maintain body composition.

## 1. Introduction

Malnutrition constitutes a major public health issue in middle- and low-income countries and is incorporated into the United Nations’ 2030 Agenda for Sustainable Development Goals [1]. Cancer is a systemic disease as it disrupts the body’s balance, even in its early stages, leading to metabolic alterations and an escalation in molecular breakdown. Initially, these abnormalities may be challenging to identify in a clinical context; nevertheless, as the illness advances, they can exacerbate and result in pronounced cancer-related cachexia [2]. Cachexia is recognized as a distinct and substantial poor prognostic indicator. It is a key factor in the deterioration of quality of life and functional impairments in cancer patients. Cancer cachexia may be associated with increased toxicity of cytotoxic treatment, frequently resulting in the earlier discontinuation of therapy [3]. Moreover, cachectic patients exhibit diminished physical functioning. A detrimental loop emerges as cachexia reduces the efficacy of cytotoxic drugs, hence promoting disease progression, which subsequently promotes the emergence of resistant cachexia [4]. This consequence results in a poor prognosis and contributes to a reduced quality of life for patients. Unfortunately, dependence on a dietary intervention alone is ineffective in these situations, necessitating further investigation to assess the efficacy of its combination with targeted medication. Consequently, it is imperative to implement strategies aimed at avoiding the emergence of advanced cachexia (stages II and III) or delaying its occurrence [5].

Sarcopenia is defined by increased levels of inflammatory cytokines, decreased serum albumin concentrations commonly observed in inflammatory conditions, and abnormal metabolism of carbohydrates, proteins, and lipids [6]. It is frequently associated with cachexia, a severe wasting syndrome widespread in several chronic diseases, which is usually irreversible unless the underlying condition ameliorates [7]. Oral nutritional supplements are often considered the optimal nutritional intervention for patients at risk of malnutrition due to their accessible and easily available advantages. The use of specialized oral nutritional supplements can enhance nutrient and energy intake orally, acting as an adjunct to compensate for insufficient regular meal consumption [8]. The data pertaining to recommendations for nutritional support therapy in patients with malignant gastrointestinal tumors, such as colorectal and gastric cancer, are controversial. Cancer patients had challenges, including inadequate food intake, reduced ability to digest and absorb nutrients, and disturbed physiological balance [9]. There are few treatment options available when a patient with gastrointestinal cancer experiences severe cachexia, there are few treatment options available, resulting in poor outcomes from anti-cancer medications [10]. Furthermore, gastrointestinal (GI) tumors demonstrate a higher mortality rate than all other cancer types [11].

This study aimed to address this matter. We conducted a randomized clinical trial (RCT) to examine the effects of oral nutrition supplementation compared with dietary advice alone on primary outcomes, such as nutritional and anthropometric outcomes, as well as secondary outcomes, such as chemotherapy tolerance.

## 2. Patients and Methods

### 2.1. Study Design

**Figure 1:**
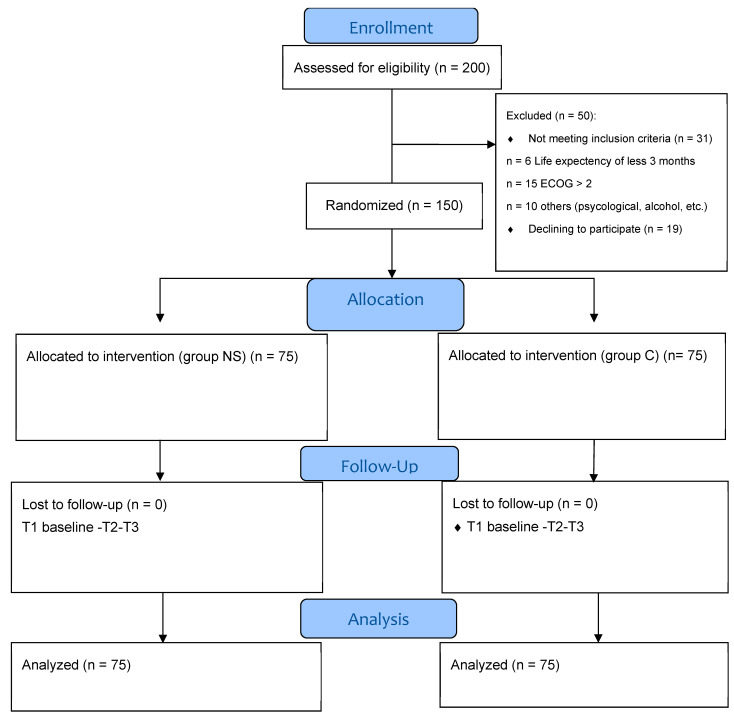
Flow diagram of this study.

This was a prospective, interventional, randomized controlled parallel open-label study (RCT) with 12 weeks of follow-up. This study aimed to ascertain how nutrition supplementation affected GIT cancer patients receiving chemotherapy (NS) compared with the control group (C). Randomization was carried out in a 1:1 ratio, using the web-based research randomizer Urbaniak and Plous [12] (Figure 1). Random sampling affords each cohort an equal probability of selection, enhancing the representativeness and generalizability of the results, while typically necessitating greater time and resources. Convenience sampling, in contrast, selects participants who are most readily accessible, resulting in a more expedient and cost-effective process; however, it is also more susceptible to bias [13]. This research was an open-label randomized controlled experiment with allocation concealment. The assigned interventions were known to both participants and investigators, although the randomization sequence was protected to avert prior knowledge of the forthcoming allocations. This methodological precaution maintained the integrity of random assignment, reduced selection bias during recruitment, and enhanced the trial’s internal validity, regardless of the lack of blinding.

This study was registered at the clinical trials registry (ClinicalTrials.gov; NCT05980624). It was conducted per the guidelines established by the International Conference on Harmonization for Good Clinical Practice and the Declaration of Helsinki. The study procedure received permission from the Research Ethical Committee of the Faculty of Medicine of Beni-Suef University (FM-BSU-REC/09072023/Rabie) and the Faculty of Pharmacy of Beni-Suef University (F PH-BSU-HREC-000525). The Integrated Guidelines for Reporting Trials (CONSORT) were followed when reporting the study outcomes. Two hundred patients were assessed for eligibility, and fifty patients were excluded from the study. One hundred and fifty patients were randomized into two groups; each group included 75 patients. Written informed consent was obtained from each participant before being enrolled in this study. This study had an intervention group with NS that was described on the manufacturer’s label for 12 weeks. The nutrition supplement was 500 kcal of a balanced powder formula daily. The balanced formula offered a complete nutritional supplement of approximately 100 kcal of energy, 3.72 g of protein, 3.27 g of fat, 13.42 g of carbohydrate, 1.01 g/100 mL of fiber, and vitamins and minerals per 100 mL. The NS group received nutrition supplements along with nutrition counselling during standard chemotherapy treatment for 6–8 cycles [14,15]. Meanwhile, the C group received standard chemotherapy plus nutrition counselling only. The study was conducted in the Oncology Department of Beni-Suef University Hospital and Fayium Oncology Centre.

### 2.2. Study Population

Patients diagnosed with any GIT cancer underwent confirmed radiological and pathological scanning procedures to assess the effectiveness of intervention via guidelines in the cancer field. Patients’ demographic information, including gender, age, and body weight, was recorded. Concurrent administration of chemotherapeutic agents, medicines, and medical conditions was carried out.

### 2.3. Inclusion Criteria

Patients with gastrointestinal (GI) cancer were included in the trial at any stage in their 1st cycle of chemotherapy. Each patient had received a confirmed cancer diagnosis, either at any stage or with metastasis, via radiological and pathological examinations or clinical evaluation. They were on neo/adjuvant chemotherapy treatment and must have been at least 18 years of age. Availability for chemotherapy treatment and subsequent medical examinations was ensured. The oral supplements provided needed to meet patients’ performance. The Eastern Cooperative Oncology Group (ECOG) performance status was limited to a maximum of two. The minimum requirement for life expectancy was at least 3 months. Each participant provided a written agreement according to the specific criteria of the Local Ethics Committee and was prepared to complete nutrition questionnaires. A thorough medical history was obtained to find any underlying causes of malnutrition or factors that raised its risk, as well as any signs of malnutrition, concentrating on a person’s precise food history, eating patterns, frequency of meals, nutrient consumption, and hydration intake. A thorough general examination was conducted that measured temperature, heart rate, and respiratory rate at admission. The daily energy target was 25 kcal/kg.

The PG-SGA indicated a comprehensive evaluation of each patient’s history and physical examination, focused on factors such as past weight loss, significant changes in nutritional consumption, loss of subcutaneous fat in two specific areas (facial and triceps), and muscle tissue loss. The symptoms and functional capacity related to nutrition were gathered from either the patient, a family member, or the patient’s medical records. The subjective aggregation of all the data yielded a score that categorized the patients as adequately nourished (SGA-A), mildly–moderately malnourished (SGA-B), or severely malnourished (SGA-C).

### 2.4. Exclusion Criteria

Patients were excluded if they exhibited allergic reactivity to the experimental chemicals or included components, if they had already been documented, or an intolerance. Patients who did not adhere to the trial requirements due to factors such as cognitive impairment, psychological or mood disorders, or alcohol addiction were excluded. This study was not suitable for individuals who were pregnant or breastfeeding. Moreover, patients were excluded when the investigator believed that a patient had a medical or psychological condition that might prevent them from participating in this study or when informed consent was not obtained. Individuals aged less than 18 years were excluded. This referred to the need for artificial nutrition support (TPN) due to a complete inability to eat normally and the inability to swallow oral nutritional supplements. Regarding ethical considerations, all the individuals included in this study were informed about the study procedures. The required administrative regulations needed to be fulfilled. Ethical approval was obtained from the Research Ethics Committee before the start of this study.

### 2.5. Primary Outcome Measures

Demographic information such as age, gender, chemotherapy type, and comorbidities was recorded for all patients at baseline (T1), as well as anthropometric measures, including body weight (kilograms) and body mass index (BMI). The BMI, weight, and height were measured at baseline and every 6 weeks up to 12 weeks of follow-up. Whole-body composition was measured using a dual X-ray absorptiometry (DEXA) scan, as well as fat mass, fat-free mass, lean body mass, BMI, and the fat mass index. All these measures were collected at baseline (T1), week 6 (T2), and week 12 (T3) of follow-up. In summary, the primary outcome was the patients’ nutritional status, as assessed by the patient-generated subjective global assessment (PG-SGA) and whole-body composition changes.

### 2.6. Secondary Outcome Measures

Biochemical laboratory data were measured every three weeks up to 12 weeks of follow-up, while lipid profile, albumin, C-reactive protein (CRP), and inflammatory mediators were assessed at baseline (T1), week 6 (T2), and week 12 (T3) to assess the nutritional and protein status as well as the disease activity. Moreover, the safety and tolerability of treatment-related adverse events, pain, and neuropathy were assessed using the Common Terminology Criteria for Adverse Events (CTCAEs) v5.0 [15]. This study evaluated the frequency of therapeutic delays and disruptions that could have an indirect impact on disease outcomes. Finally, the survival and response were reported after 6 months of follow-up. The results are presented in Supplementary Table S3.

### 2.7. Statistical Analysis

#### 2.7.1. Sample Size Calculation

Based on findings reported in the literature, the sample size was 150 patients overall to guarantee 80% power and account for type I error, with a probability of 5%. The statistical software G Power, version 3, was used to calculate the sample size [16]. We anticipated that the 12-week nutrition supplement intervention would prevent a weight loss of 1.1 kg (2% of body weight) based on a previous study to detect an effect size of 2% using a two-sided alpha error of 5%, assuming a normal distribution with a standard deviation of 2.2 [17]. Convenience sampling was used, which included all patients who had received chemotherapy for GI cancer.

#### 2.7.2. Descriptive and Inferential Statistics

Statistical analyses were conducted using IBM SPSS Statistics, version 28, ensuring robust computations and appropriate visualization of the results. Thereafter, the data were processed and tabulated. A frequency distribution was generated, along with its corresponding percentages. Additionally, descriptive statistics, such as the mean and standard deviation, were produced. Significance was attributed to *p*-values below 0.05. Linear regression was applied to determine the relationships between independent variables and the dependent variable. Model assumptions for multiple comparison *p*-values were determined after Bonferroni corrections. For repeated measurements over time, repeated-measures ANOVA was performed to evaluate the main effects of time and group, as well as interaction effects between these factors on the outcome variables. Pairwise group differences were examined using post hoc comparisons with Bonferroni-adjusted *p*-values to account for multiple comparisons following ANOVA. *p*-values were determined after Bonferroni correction by multiplying *p* by the correction term (M), which equaled the number of possible pairs and the number of pairwise tests. *p* corrected equaled p*m [18]. Corrected *p*-values were obtained using the Bonferroni adjustment for post hoc pairwise comparisons by multiplying each *p*-value by the number of pairwise comparisons (m); thus, the significance level was adjusted from α = 0.05 to control for multiple testing. Intention-to-treat (ITT) analysis was conducted in the statistical analysis of the results.

## 3. Results

### 3.1. Baseline Clinical Characteristics

The subjects were 51.31 years old on average and received either adjuvant or neoadjuvant chemotherapy (Table 1). All subjects were in the first cycle of chemotherapy at the time of study. The NS and C groups demonstrated no significant differences in terms of age, weight, BMI, diagnosis, recurrence, clinical stage, number of lymph nodes, comorbidities, and chemotherapy treatment. The mean adherence was 0.847 ± 0.1803 compared to the oral nutrition supplements in the NS group. The compliance with the intervention for the NS group was 84.7%.

### 3.2. Analysis of Change in Whole-Body Composition

The data were expressed as means ± standard deviations. The time effect, group effect, and the interaction between time and group were determined. Differences in values between week 0 (T1), week 6 (T2), and week 12 (T3) within the group were determined. Supplementary Materials Table S1 was compared between both groups at each time point.

Table 2 presents the longitudinal comparison of various anthropometric and whole-body composition parameters between the C group and NS group using one-way repeated-measures ANOVA. Statistically significant time effects were observed for weight (*p* = 0.017), BMI (*p* = 0.02), fat-free mass (FFM) (*p* = 0.001), lean mass (*p* = 0.001), lean mass/height^2^ (*p* = 0.025), the APP appendicular lean mass index (*p* = 0.04), and the subcutaneous adipose tissue area (SATA) (*p* = 0.018), indicating within-group changes over time. Weight and BMI significantly increased over time in the NS group (*p* < 0.001 and 0.005 for weight, and *p*= 0.001 and *p*= 0.006 for BMI, particularly between T1 (week 0)–T2 (week 6) and T1 (week 0)–T3 (week 12). Conversely, no significant changes were seen in the C group. A significant group-by-time interaction was noted for weight (*p* = 0.044), lean mass/height^2^ (*p* = 0.007), and the FMI (*p*= 0.023), suggesting that the nutritional supplement intervention in the NS group had a distinct temporal impact compared with the C group. Moreover, Table 3 shows that there was a substantial difference in weight change, with the BMI in the NS group showing a consistent rise throughout the course of the 12-week follow-up.

Significant increases in the FFM and lean mass were observed in the NS group across all timepoints (*p* < 0.001). Moreover, the group-by-time interaction was statistically significant for lean/height^2^ (*p* = 0.007). Fat mass (FM) in grams did not show significant group-by-time effects in either group *p* = 0.063; however, FM showed a statistically significant improving trend in the NS group (T2–T3; *p* = 0.034), with a non-significant trend at the end of 12 weeks (T1–T3; *p* = 0.11) in the NS group. The FMI (fat per height squared) group-by-time interaction was statistically significant between both groups, confirming the trend showing an increase in the NS group after 12 weeks of intervention, whereas there was a decrease in the C group. Briefly, there was a significant, steady increase in the group effect at 12 weeks in the NS group. Moreover, the FMI showed a significant group-by-time effect, with a steady decrease in the C group’s FMI (*p* = 0.023 *).

Additionally, the group effects for FM, the FMI, the visceral adipose tissue area (VATA), visceral fat, and the VAT volume (VATV) were significant (*p* < 0.05), with less loss in the NS, suggesting overall differences in body composition attributable to the intervention.

The changes in the VAT parameters (VATA, visceral fat, and VATV) were not statistically significant over time within either group, nor were interaction effects observed. However, the group differences were significant, indicating baseline or persistent between-group differences. Significant time (*p* = 0.018) and group (*p* < 0.001) effects were found for the SATA, yet the within-group changes did not reach significance post hoc.

In summary, the NS group maintained a lean body mass that significantly differed from the baseline. Moreover, the lean mass index showed a statistically significant group and group-by-time effect with maintained lean body mass in the NS group at 12 weeks.

Multivariate mixed linear regression showed that patients in the NS group were more likely to reach a 1.6 times higher weight (*p* = 0.01) and a 0.623 times higher BMI (*p* = 0.025) after 12 weeks compared with those in the C group, adjusted to the baseline NRS, baseline weight, gender, and age (Table 3).

The results in Table 3 represent a linear regression analysis examining the predictors of DEXA (dual X-ray absorptiometry) scan outcomes after 12 weeks. The model identified several significant factors, including multivariate analysis adjusted to age and the baseline NRS. Weight and BMI were the most important outcomes in the DEXA scan. The primary outcomes in this study are presented in Table 3.

The model identified several significant factors influencing weight outcomes. Receiving nutritional supplements in the NS group was associated with a statistically significant increase in weight (β = 1.652; *p* = 0.01) and an additional increase in BMI compared with the control group (B = 0.623; *p* = 0.025), suggesting a beneficial effect of the intervention. Age showed a significant negative association with weight change (β = –0.075; *p* = 0.001), indicating that older patients tended to gain less or lose more weight over the follow-up period. The baseline nutritional risk score (NRS) also had a significant inverse relationship with weight change (β = –0.695; *p* < 0.001), implying that patients with higher nutritional risk at baseline experienced greater weight loss. In contrast, sex (female) and baseline weight were not significantly associated with weight change (*p* = 0.262 and *p* = 0.351, respectively). The intercept of the model was also statistically significant (β = 5.522; *p* = 0.014), indicating a positive baseline weight trajectory when all predictors were held at zero. Overall, the findings highlight the clinical value of targeted nutritional support and the need to consider age and baseline nutritional risk in managing weight outcomes among cancer patients.

### 3.3. PG-SGA Patient-Generated Subjective Global Assessment

The PG-SGA score and grade used to evaluate nutritional status were compared between the two study groups, as shown in Table 4. The PG-SGA scores at baseline were 12.37 and 10.68 in the NS and C groups, respectively (Table 4). The NS group lowered PG-SGA scores from 12.37 ± 3.8 to 6.12 ± 2.69 (*p* < 0.001) at 12 weeks after intervention, while there was no change in the C group.

Linear regression indicated the PG-SGA scores were significantly improved depending on the study duration in the NS group, as shown in Table 5, with a detailed table in Supplementary Materials Table S2.

Patients in the NS group were more likely to obtain a 5.713 times lower PG-SGA score after 12 weeks compared with those in the C group, adjusted to the baseline NRS, baseline PG-SGA, gender, and age, with *p* < 0.001, as shown in Table 5.

Meanwhile, the NS and C groups showed significant changes in PG-SGA grades, with increases in grade A (16% to 60% and 31.6% to 78.67%, respectively) for the NS group compared with the C group, which increased in the C grade (24%, 25.33%, and 41.34%) (Table 6). Conversely, clinical outcomes such as survival and response after 6 months of follow-up reported no significance in both groups, as presented in Supplementary Materials Table S3.

### 3.4. Analysis of Biochemical Nutritional Markers

There was a statistically significant difference in albumin serum levels at 12 weeks between both groups (difference T3–T1; *p* < 0.001). Meanwhile, total protein changed non-significantly between the groups (Table 7). Other nutritional biomarkers, including TGs, cholesterol, total protein, and CRP, showed statistically non-significant changes in both study groups (Table 8).

### 3.5. Analysis of Adverse Effects and Therapy Delay

Statistically significant differences in adverse events related to pain (*p* = <0.001), grade 3 neuropathy (*p* = 0.002), hand–foot syndrome (*p* = 0.027), and the need for liver support (*p* = 0.018) appeared in the C group; moreover, the NS group showed less delay in therapy and dose modification (*p* < 0.001), as presented in Table 9 and Table 10.

## 4. Discussion

There is limited evidence to promote dietary supplementation during oncological treatment, despite strong data suggesting that malnutrition is a predictive factor for poor clinical outcomes after chemotherapy. It is necessary to investigate the contributing factors that lead to oral intake deficiencies. These factors may be inherent (such as anorexia), connected to chemotherapy, or the result of inadequate nutritional support, either in terms of quality or quantity. A well-designed RCT is urgently needed to examine how nutritional interventions affect treatment toxicities, chemotherapy tolerance, therapy delay, and nutritional outcomes, and we offer suggestions for how these studies should be planned. More research should be undertaken on the best times and lengths for nutritional interventions, as well as methods to increase adherence to dietary advice.

This study was a randomized clinical trial conducted to evaluate the efficacy of a 12-week intervention using a balanced oral nutrition supplement formula for nutritional outcomes. To the best of our knowledge, this is the first multicenter study in the MENA region to address this issue.

All measures of body composition, including body weight, BMI, lean mass, and fat mass, significantly increased among patients in the NS group, whereas fat mass significantly decreased in the C group. Conversely, the C group demonstrated a reduction in fat mass and visceral fat mass. Changes in body composition revealed that enhanced oral supplements maintained lean body mass and body weight in cancer patients, aligning with our findings showing that the NS group greatly preserved the lean height index [19]. In addition, NS positively affected fat mass, consistent with other research [20]. The NS group exhibited preserved fat-free mass, lean muscle mass, and fat mass in patients receiving chemotherapy. Approximately 90% of the body’s total energy source was derived from fat, which plays a crucial role in sustaining weight and nutritional status in cachectic cancer patients, as fat loss results in weight reduction in this population [19]. In progressing cancer patients, an increased rate of fat mass reduction correlates with a notable decline in the survival rate [19].

There was no statistically significant difference in the appendicular lean mass index between the two groups; however, the NS group exhibited a rise and maintenance of AppLM at week 6, indicating that greater cycle chemotherapy adversely affects patient outcomes. Previous studies demonstrated that early nutritional management during chemotherapy minimized the risk of malnutrition and enhanced the rate of treatment completion [21,22]. Chemotherapy alterations, such as delays, dosage reductions, or termination, were significantly reduced in the NS group, aligning with previous studies [14,23]. Comparable findings were also seen in previously published meta-analyses [24,25].

Despite the distinct nature of malnutrition caused by cancer cachexia compared with other disease-related or age-related malnutrition, nutrition screening methods, particularly designed for cancer patients, are lacking. PG-SGA is the most generally recognized technique for evaluating the nutritional status of cancer patients [26]. The PG-SGA technique can precisely represent the fluctuating nutritional status of patients, as it considers acute weight fluctuations with intermediate or chronic weight changes, unlike other nutritional assessment tools [27]. This study indicated a considerable enhancement in PG-SGA scores for the NS groups. According to these findings, the NS group’s nutritional status improved by the trial’s completion, but the C group’s did not, as shown by the multivariate mixed linear regression, which is consistent with prior research [19,28]. The PG-SGA score significantly increases in the chemotherapy control group, where patients had chronic lower consumption, leading to a progressive decline in nutritional status and an elevated risk of malnutrition [29]. The PG-SGA score was improved in the NS group. This might impact treatment completion, with fewer adverse effects and therapy delays, which was proven in our research and agrees with the latest meta-analyses [30,31].

However, nutritional supplements did not substantially influence the status of nutritional biomarkers, including total protein, cholesterol, CRP, LDL, and HDL levels [32,33]. This may be because individuals undergoing chemotherapy experience unstable metabolic issues. Similarly, a previous study on esophageal cancer patients undergoing chemotherapy revealed no significant changes in total serum protein or total cholesterol levels [34,35].

This study additionally employed nutritional biomarkers to assess nutritional status. Our study had similar biomarker results to a previously published comprehensive review that examined how well nutrition supplements work to improve the nutritional status of cancer patients [36,37]. This study revealed a significant difference favoring the nutrition group in the albumin biomarker, with patients in the nutrition group exhibiting a 0.784 times higher albumin level after 12 weeks compared with the control group, adjusted for the baseline NRS, gender, and age, using multivariate mixed linear regression. Moreover, a previous study obtained similar results to ours, finding no statistically significant difference in other biochemical markers in both groups, such as the complete lipid profile, CRP, and total protein [14,38,39]. Comparable outcomes were observed when patients received home enteral feeding, as previously published [40]. The results show that NS possesses therapeutic potential and may reduce the nutritional risk for patients with GIT cancer. NS is a crucial non-invasive nutritional support approach for patients able to consume food but whose intake fails to meet their total nutritional requirements. Moreover, chemotherapy may lead to the deterioration of cancer patients’ body systems [41,42], thus impairing their ability to synthesize albumin. Albumin is a major component of human plasma proteins, crucial for maintaining essential physiological functions, including plasma colloid osmotic pressure and transport, while also enhancing the tolerance of enteral nutrition [40,43]. An evaluation of the correlation between albumin levels and survival time demonstrated that albumin serves as an independent prognostic factor for overall survival time and is correlated with the patient’s total survival time. Therefore, it is important to closely monitor the biochemical markers of cancer patients throughout chemotherapy, as these indicators are vital for evaluating nutritional risk and determining the continuation of treatment [41,44].

In this study, while most biochemical indicators did not show significant differences, except for albumin levels at all time points, the improvement in albumin levels in the NS group was superior to that in the control group, highlighting the crucial role of NS in chemotherapy. However, in contrast with our findings, prior studies indicated that NS intervention did not provide a beneficial effect on biochemical markers [19,34]. This may have resulted from varying compliance and adherence in the NS group. Finally, the significance observed in T2-T3 can be attributed to the difference in compliance or adherence to nutrition support supplements over time [45].

This study had certain limitations. Initially, while patient compliance exhibited greater variability in this trial, the findings may have been influenced by the limited sample size, despite NS positively affecting the nutritional condition of GIT cancer patients. Secondly, practical objective factors that may have compromised individuals across groups hindered the implementation of a double-blind design in this investigation. Despite the PG-SGA being utilized in this study in full accordance with the operational requirements recommended by the American Academy of Nutrition and Dietetics, the linguistic validity of the Arabic version of the PG-SGA remains uncertain [46]. Finally, there was a potential selection bias or heterogeneity of cancer types and chemotherapy regimens, which might have affected the results.

## 5. Conclusions

This study demonstrated that the administration of oral nutrition supplements may have the potential to maintain weight, BMI, lean body mass, and fat mass in individuals who are at risk of malnutrition by enhancing dietary outcomes. Furthermore, it may improve chemotherapy tolerance in patients with GIT cancer, enabling the completion of treatment and a reduction in therapy delays. These findings highlighted the potential effectiveness of the nutritional program in modifying body composition over time.

## Figures and Tables

**Table 1:** Baseline characteristics of patients.

Variable	C Group		NS Group				*t*-Test	*p*-Value
	Mean	SD	Mean		SD			
Age	50.05	11.95	52.57		12.55		−1.26	0.21
Weight	61.32	14.58	59.42		12.63		−0.85	0.40
BMI	24.53	4.91	23.34		4.94		−1.49	0.14
**Variables**			**Control (n = 75)**		**Nutrition (n = 75)**		**Test Value**	***p*** **-Value**
			**No.**	**%**	**No.**	**%**		
Staging	2		49	65.33	55	73.33	1.29	0.53
	3		22	29.33	16	21.33		
	4		4	5.33	4	5.33		
T	0		2	2.67	0	0.00	3.52	0.32
	2		12	16.00	18	24.00		
	3		47	62.67	42	56.00		
	4		14	18.67	15	20.00		
N	0		22	29.33	25	33.33	14.4	0.002
	1		29	38.67	23	30.67		
	2		11	14.67	25	33.33		
	3		13	17.33	2	2.67		
M	0		63	84.00	59	78.67	6.16	0.15
	1		3	4.00	7	9.33		
	2		9	12.00	7	9.33		
No. of LN, median (IQR)			2	0–9	2	0–6	−0.88	0.38
Recurrence	Yes		6	8.00	5	6.67	0.09	0.75
	No		69	92.00	70	93.33		
Therapy	0		10	13.33	6	8.00	18.06	<0.001
	1		32	42.67	57	76.00		
	2		19	25.33	8	10.67		
	3		14	18.67	4	5.33		
Comorbidities	HTN		12	16.00	5	6.67	3.25	0.071
	DM		15	20.00	15	20.00	0.00	1.00
Chemotherapy type	0		31	41.33	32	42.67	10.87	0.09
	1		12	16.00	8	10.67		
	2		8	10.67	8	10.67		
	3		8	10.67	9	12.00		
	4		6	8.00	6	8.00		
	5		6	8.00	0	0.00		
	6		4	5.33	12	16.00		
Diagnosis	Colorectal		43	57.33	38	50.67	0.67	0.74
	Gastroesophageal		14	18.67	16	21.33		
	Pancreatic		18	24.00	21	28.00		

Therapy: chemo = 0/chemosurgery = 1/chemo-radio = 2/chem/surgery/radio = 3; chemotherapy type 0 = XELOX 1 = GEMZAR/XELODA 2 = FOLFOX 3 = FOLFOXIRI 4 = GEMZAR/CISPLATIN 5 = FOLFIRI 6 = XELODA CAMPTO. BMI—body mass index; lymph node N = 0/1/2/ > 3; metastasis M 0/1/X = 2.

**Table 2:** Post hoc pairwise comparison between both groups over time using one-way repeated-measures ANOVA.

	Repeated-Measures ANOVA		Time		C Group		NS Group	
					(n = 75)		(n = 75)	
	Source	*p*-Value			Mean ± SE	*p*-Value	Mean ± SE	*p*-Value
Weight ^¥^	Time	<0.017 *	T1	−T2	0.146 ± 0.24	1.00	−0.835 ± 0.21	≤0.001 *
	Group	0.6		−T3	−0.154 ± 0.426	1.00	−1.27 ± 0.39	0.005 *
	Time * gp	0.044 *	T2	−T3	−0.3 ± 0.32	1.00	−0.432 ± 0.27	0.32
BMI ^¥^	Time	<0.02 *	T1	−T2	0.144 ± 0.10	0.418	−0.327 ± 0.09	0.001 *
	Group	0.26		−T3	−0.10 ± 0.193	1.00	−0.48 ± 0.15	0.006 *
	Time * gp	0.059	T2	−T3	−0.244 ± 0.169	0.461	−0.16 ± 0.11	0.435
FFM ^¥^	Time	<0.001 *	T1	−T2	−0.754 ± 0.31	0.056	−0.683 ± 0.13	<0.001 *
	Group	0.038 *		−T3	−0.941 ± 0.407	0.071	−0.79 ± 0.19	<0.001 *
	Time * gp	0.91	T2	−T3	−0.187 ± 0.355	1.00	−0.1 ± 0.18	1.00
FM ^¥^	Time	0.08	T1	−T2	0.603 ± 0.266	0.08	−0.127 ± 0.19	1.000
	Group	0.002 *		−T3	0.209 ± 0.338	1.00	−0.55 ± 0.26	0.11
	Time * gp	0.063	T2	−T3	−0.393± 0.246	0.344	−0.42 ± 0.17	0.034 *
FMI ^¥^	Time	0.156	T1	−T2	0.28 ± 0.11	0.045 *	−0.038 ± 0.08	1.00
	Group	0.004 *		−T3	0.2 ± 0.154	0.625	−0.286 ± 0.14	0.116
	Time * gp	0.023 *	T2	−T3	−0.082 ± 0.1	1.00	−0.249 ± 0.117	0.112
Lean ^¥^	Time	<0.001 *	T1	−T2	−0.44 ± 0.25	0.22	−0.697 ± 0.13	<0.001 *
	Group	0.053		−T3	−0.762 ± 0.335	0.078	−0.73 ± 0.19	<0.001 *
	Time * gp	0.65	T2	−T3	−0.318 ± 0.342	1.00	−0.034 ± 0.17	1.000
Lean/H^2 ¥^	Time	0.025 *	T1	−T2	0.045 ± 0.17	1.000	−0.538 ± 0.12	<0.001 *
	Group	0.03 *		−T3	−0.14 ± 0.147	1.000	−0.265 ± 0.072	0.001 *
	Time * gp	0.007 *	T2	−T3	−0.186 ± 0.15	0.645	0.28 ± 0.13	0.119
App lean I ^¥^	Time	0.04 *	T1	−T2	0.210 ± 0.11	0.208	−0.219 ± 0.06	0.001 *
	Group	0.56		−T3	0.444 ± 0.234	0.185	0.18 ± 0.19	1.000
	Time * gp	0.26	T2	−T3	0.234 ± 0.25	1.00	0.399± 0.21	0.195
VATA ^¥^	Time	0.78	T1	−T2	6.216 ± 8.55	1.000	−4.35± 5.81	1.00
	Group	0.013 *		−T3	0.742 ± 8.858	1.000	−2.493 ± 6.28	1.00
	Time * gp	0.48	T2	−T3	−5.474 ± 4.076	0.55	1.856 ± 5.21	1.00
Visceral ^¥^	Time	0.30	T1	−T2	107.69 ± 61.9	0.259	−2.73 ± 46.72	1.00
	Group	0.007 *		−T3	52.83 ± 65.57	1.000	13.485 ± 41.502	1.00
	Time * gp	0.26	T2	−T3	−54.86 ±30.7	0.234	16.217 ± 34.46	1.00
VATV ^¥^	Time	0.58	T1	−T2	85.383 ±63.71	0.553	−80.798 ±62.98	0.611
	Group	0.003 *		−T3	16.413 ±64.48	1.000	−75.15± 43.734	0.27
	Time * gp	0.099	T2	−T3	−68.96 ± 31.28	0.092	5.648 ± 38.018	1.00
SATA ^¥^	Time	0.018 *	T1	−T2	9.932 ± 5.73	0.262	7.899 ± 4.051	0.165
	Group	<0.001 *		−T3	4.11 ± 5.682	1.000	−3.09 ± 5.022	1.00
	Time * gp	0.572	T2	−T3	−5.817 ± 3.744	0.374	−10.987 ± 5.199	0.114

BMI—body mass index; FM—fat mass; FFM—fat-free mass; lean—lean body mass; lean mass index (kg/H^2^) APP—appendicular lean mass index (kg/H^2^); VATA—visceral adipose tissue area (cm^2^); visceral adipose tissue (grams), VATV—visceral adipose tissue volume (cm^3^); SATA—subcutaneous adipose tissue area (cm^2^). T1 (week 0), T2 (week 6), and T3 (week 12). ^¥^: The Greenhouse–Geisser test was used, as Mauchly’s test of sphericity result was significant; *: significant *p*-value.

**Table 3:** Prediction of weight change and BMI after 12 weeks using linear regression among GIT cancer patients.

Outcome	Univariate Linear Regression				Multivariate Linear Regression			
	Beta	SE	*p*-Value	95% CI	Adjusted Beta	SE	*p*-Value	95% CI
Weight	1.113	0.578	0.056	−0.02, 2.25	1.652	0.63	0.01 *	0.42, 2.89
BMI	0.383	0.245	0.12	−0.1, 0.86	0.623	0.275	0.025 *	0.08, 1.16

Beta—regression coefficient; SE—standard error; *—significant *p*-value at 0.05 level; CI—confidence interval at 95%. The multivariate regression weights were adjusted for sex, age, the baseline NRS, and the baseline measured parameter.

**Table 4:** The changes in patient-generated subjective global assessment (PG-SGA) scores between the C and NS groups.

Parameter	C Group		NS Group		*p*-Value
	Mean	SD	Mean	SD	
PGSGA 1	10.68	3.48	12.37	3.80	0.005 *
PGSGA 2	11.21	4.35	6.92	3.04	<0.001 *
PGSGA 3	11.39	5.12	6.12	2.96	<0.001 *
Difference T2–T1	0.53	3.90	−5.45	3.68	<0.001 *
Difference T3–T2	0.17	3.11	−0.80	2.10	0.026 *
Difference T3–T1	0.7067	3.69724	−6.2533	3.44098	<0.001 *

A negative value indicated a decrease in the difference. A *t*-test was used at a significance level of 0.05. T1 (week 0), T2 (week 6), and T3 (week 12). * significance level at *p* value ≤ 0.05

**Table 5:** Linear regression to predict change in PG-SGA after 12 weeks.

Outcome	Univariate Linear Regression				Multivariate Linear Regression			
	Beta	SE	95% CI	*p*-Value	Adjusted Beta	SE	95% CI	*p*-Value
PGSGA	−5.987	0.619	−7.2, −4.77	<0.001 *	−5.713	0.747	−7.18, −4.25	<0.001

* significance level at *p* value ≤ 0.05

**Table 6:** The change in the patient-generated subjective global assessment (PG-SGA) grade ratio between the C and NS groups.

Variables	C Group (n = 75)		NS Group (n = 75)		Test Value	*p*-Value	
	No.	%	No.	%			
PGSGA T1	A	17	22.67	12	16	3.37	0.186
	B	40	53.33	35	46.67		
	C	18	24	28	37.33		
PGSGA T2	A	17	22.67	45	60	25.03	<0.001 *
	B	39	52	26	34.67		
	C	19	25.33	4	5.33		
PGSGA T3	A	25	33.33	59	78.67	40.00	<0.001 *
	B	19	25.33	14	18.67		
	C	31	41.34	2	2.66		

* significance level at *p* value ≤ 0.05.

**Table 7:** Changes in nutritional biochemical markers between groups.

Variable	C Group			NS Group			*p*-Value		
	Mean ± SD			Mean ± SD					
	T1	T2	T3	T1	T2	T3	a	b	c
CRP	15.58 ± 14.77	11.49 ± 13.4	13.02 ± 14.55	16.71 ± 20.25	13.76 ± 16.74	17.87 ± 20.67	0.698	0.362	0.099
Diff. T3–T1	−2.56 ± 17.14			1.16 ± 20.86			0.235		
Albumin	4.17 ± 0.71	4.14 ± 0.5	3.71 ± 0.71	3.98 ± 0.74	4.12 ± 0.66	4.16 ± 1	0.123	0.824	0.002 *
Diff. T3–T1	−0.46 ± 1.04			0.17 ± 0.94			<0.001 *		
TGs	123.38 ± 55.78	123.31 ± 41.83	120.9 ± 34.82	128.63 ± 76.3	119.63 ± 70.26	117.52 ± 68.27	0.631	0.698	0.704
Diff. T3–T1	−2.49 ± 58.75			−11.11 ± 45.42			0.316		
Cholesterol	175.64 ± 48.35	161.93 ± 28.95	167.61 ± 21.19	160.56 ± 46.26	161.66 ± 50.41	159.89 ± 48.76	0.053	0967	0.211
Diff. T3–T1	−8.03 ± 45.49			−0.68 ± 44.49			0.321		
HDL	40.76 ± 6.63	42.16 ± 7.55	41.48 ± 5.73	36.76 ± 12.89	42.02 ± 8.96	40.95 ± 7.89	0.018 *	0.918	0.639
Diff. T3–T1	0.71 ± 8.91			4.19 ± 16.43			0.11		
LDL	108.81 ± 44.92	94.76 ± 28.94	101.26 ± 24.1	97.43 ± 37.29	89.39 ± 42.43	95.82 ± 45.97	0.094	0.367	0.366
Diff. T3–T1	−0.7.54 ± 43.75			−1.6 ± 44.95			0.414		
VLDL	24.67 ± 11.16	25.01 ± 8.33	24.49 ± 7.88	25.88 ± 15.31	24.98 ± 14.71	23.41 ± 13.61	0.582	0.99	0.553
Diff. T3–T1	−0.18 ± 12.38			−2.47 ± 9.07			0.199		
T protein	8.71 ± 1.24	8.64 ± 1.48	8.08 ± 1.55	9.08 ± 1.4	8.3 ± 1.13	8.39 ± 1.24	0.086	0.11	0.177
Diff. T3–T1	−0.62 ± 1.81			−0.69 ± 1.71			0.828		

* significance level at *p* value ≤ 0.05.

**Table 8:** Linear regression models to predict changes in the following outcomes.

Outcomes	Univariate Linear Regression				Multivariate Linear Regression			
	Beta	SE	95% CI	*p*-Value	Adjusted Beta	SE	95% CI	*p*-Value
CRP	3.722	3.12	−2.39, 9.84	0.234	6.291	3.632	−0.83, 13.41	0.085
Albumin	0.631	0.162	0.31, 0.95	<0.001 *	0.784	0.183	0.43, 1.14	<0.001 *
TGs	−8.62	8.58	−25.44, 8.2	0.316	−3.718	9.258	−21.86, 14.43	0.689
Cholesterol	7.35	7.385	−7.12, 21.82	0.321	0.624	7.588	−14.25, 15.5	0.94
HDL	3.474	2.158	−0.76, 7.7	0.110	0.529	1.492	−2.4, 3.45	0.72
LDL	5.94	7.243	−8.26, 20.14	0.414	−2.22	7.664	−17.24, 12.8	0.77
vLDL	−2.29	1.77	−5.76, 1.18	0.199	−1.494	1.932	−5.28, 2.29	0.44
Tot. protein	−2.287	1.77	−5.76, 1.18	0.199	0.503	0.3	−0.09, 1.09	0.096

Beta—regression coefficient; SE—standard error; *—significant *p*-value at 0.05 level; CI—confidence interval at 95%. The multivariate regression weights were adjusted for sex, age, the baseline NRS, and the baseline measured parameter.

**Table 9:** Side effects in the two study groups.

Variable		C Group (n = 75)		NS Group (n = 75)		Test Value	*p*-Value
Fatigue		36	48.00	40	53.33	0.43	0.514
Smell change		2	2.67	4	5.33	0.69	0.68
Pain	Non	24	32.00	33	44.00	17.8	<0.001 *
	G1	35	41.33	40	53.33		
	G2	11	14.67	2	2.67		
	G3	5	6.67	0	0.00		
Neuropathy	Non	44	58.67	51	68.00	11.88	0.002 *
	2	20	26.67	24	32.00		
	3	11	14.67	0	0.00		
Hand–foot		14	18.67	5	6.67	4.88	0.027 *
Vision disturbance		3	4.00	5	6.67	0.528	0.719
Vertigo		5	6.67	6	8.00	0.098	0.75
Headache		16	21.33	11	14.67	1.13	0.29
Liver support		9	12.00	1	1.33	6.86	0.018*
Anxiety		37	49.33	28	37.33	2.43	0.12

* significance level at *p* value ≤ 0.05.

**Table 10:** Delay in therapy and dose reduction in the two study groups.

Variable		C Group (n = 75)		NS Group (n = 75)		Test Value	*p*-Value
Need for dose reduction		22	29.33	5	6.67	13.05	<0.001 *
Frequency	0	53	70.67	70	93.33	13.52	<0.001 *
	1	19	25.33	5	6.67		
	2	3	4.00	0	0.00		
Therapy delay		47	62.67	25	33.33	12.93	<0.001 *
Frequency	0	28	37.33	50	66.67	17.25	<0.001 *
	1	25	33.33	18	24.00		
	2	15	20.00	7	9.33		
	3	7	9.33	0	0.00		

* significance level at *p* value ≤ 0.05.

## Data Availability

The data will be released upon reasonable request from the corresponding author.

## Supplementary Materials

The following supporting information can be downloaded at: https://www.mdpi.com/article/10.3390/pharmaceutics17111443/s1, Supplementary Table S1: Comparison between control and nutrition support group in terms of mean Body composition at baseline w0, w6 and w12 each time point; Supplementary Table S2: Detailed Linear regression to predict change in PG-SGA after 12 weeks; Supplementary Table S3: Response and survival in 6 months follow up in both groups there was no significant.
