# Supplementary material for: Impact of Oral Nutrition Supplements in Gastrointestinal Cancer Patients: A Randomized Controlled Trial
Source: Pharmaceutics. 2025 Nov 8;17(11):1443. doi: 10.3390/pharmaceutics17111443 (PMC12655240; doi:10.3390/pharmaceutics17111443)
Supplement: Supplementary file 1 [file pharmaceutics-17-01443-s001.zip › pharmaceutics-3864268-supplementary.pdf]

pharmaceutics

MDPI

Table S1: Comparison between control and nutrition support group in terms of mean Body composition at baseline w0 , w6 and w12 each time point.

Varia-ble	C group<br>Mean± SD			NS group<br>Mean±SD			p-value		
	T1	T2	T3	T1	T2	T3	a	b	c
Wight	61.32±14.58	61.17±15.18	61.47±15.97	59.42±12.63	60.25±12.62	60.69±13.3	0.40	0.69	0.75
Diff T3-T1	0.15±3.69			1.27±3.39			0.06		
BMI	24.53±4.91	24.39±4.86	24.63±5.31	23.34±4.94	23.66±4.92	23.82±5.16	0.14	0.36	0.34
Diff T3-T1	0.10±1.67	0.12		0.48±1.31			0.12		
FFM	37.57±10.07	38.32±10.25	38.51±10.41	40.81±8.36	41.49±8.3	41.60±8.63	0.03*	0.04*	0.05*
Diff T3-T1	0.94±3.53			0.79±1.66			0.73		
Lean mass	35.77±9.43	36.22±9.75	36.53±9.83	38.51±7.99	39.20±7.92	39.24±8.17	0.06	0.04*	0.07
Diff T3-T1	0.76±2.9			0.73±1.64			0.94		
FM	23.48±8.68	22.88±8.47	23.27±9.04	18.63±8.02	18.76±7.81	19.18±8.32	0.00*	0.00*	0.01*
Diff T3-T1	-0.21±2.93			0.55±2.24			0.08		
APP Lean	6.89±2.17	6.68±2.19	6.45±1.47	6.52±1.77	6.74±1.96	6.34±1.01	0.25	0.87	0.59
Diff T3-T1	-0.44±2.03			-0.18±1.63			0.38		
VATA	153.02±96.9	146.80±89.3	152.27±84.3	118.14±70.1	122.49±82.2	120.63±57.5	0.01*	0.09	0.01*

Diff T3-T1	-0.74±76.71			2.49±54.38			0.77		
Vis-ceral	930.08±722.7	822.39±689.86	877.25±712.57	638.78±437.62	641.51±519.29	625.30±399.45	0.00*	0.07	0.01*
Diff T3-T1	-52.83±576.87			-13.48±359.42			0.61		
VATV	950.21±721.75	864.83±724.92	933.80±753.76	591.86±416.24	672.66±547.05	667.01±412.77	0.00	0.07	0.01*
Diff T3-T1	-16.41±558.48			75.15±378.75			0.24		
SATA	85.65±54.6	75.72±45.64	81.54±40.17	59.72±32.59	51.82±28.6	62.80±53.32	0.00*	0.00*	0.02*
Diff T3-T1	-4.12±49.21			3.09±43.49			0.34		

a* significance at T1 Baeline between both groups b* significance at T2 6 weeks between both groups c* significant at T3 12 weeks between both groups, BMI body mass index, FM fat mass, FFM fat free mass, Lean lean body mass APP appndiclar lean mass index kg/H^{2,} VATA visceral adipose tissue area cm^2 . Visceral adipose tissue gram, VATV visceral adipose tissue volume cm^3, SATA subcutaneous adipose tissue area cm^2

Table S2 Detailed Linear regression to predict change in PG-SGA after 12 weeks

	Beta	Std. Error	t-test	p-value
(Constant)	-3.365	2.745	-1.226	0.222 95% CI 7.18, -4.25
NS group	-5.713	0.747	-7.65	<0.001*
Female sex	-1.583	0.647	-2.446	0.016*
Age	0.004	0.024	0.161	0.872
Adherence to protocol	0.167	2.243	0.074	0.941
Baseline NRS T1	0.068	0.223	0.306	0.76
Baseline PGSGA T1	0.311	0.091	3.422	0.001*

Table S3 Response and survival in 6 months follow up in both groups there was no significant.

	C group(n=75)		NS group (n=75)		T-test value	p-value
	No.	%	No.	%		
Response						
Complete response	25	33.33	35	46.67	3.65	0.302
Partial response	25	33.33	19	25.33		
Stable disease	5	6.67	8	10.67		
Stationary disease	17	22.67	13	17.33		
Survival						
Alive	71	94.67	65	86.67	2.84	0.092
Died	4	5.33	10	13.33		

Chi-square test was conducted. *: significant p-value at 0.05 level.
